# Applying an Archetype-Based Approach to Electroencephalography/Event-Related Potential Experiments in the EEGBase Resource

**DOI:** 10.3389/fninf.2017.00024

**Published:** 2017-04-06

**Authors:** Václav Papež, Roman Mouček

**Affiliations:** ^1^Department of Computer Science and Engineering, University of West Bohemia, Plzeň, Czech Republic; ^2^New Technologies for the Information Society (NTIS), University of West Bohemia, Plzeň, Czech Republic

**Keywords:** electroencephalography, event-related potentials, ontology, openEHR, archetype, odML, EHR

## Abstract

**Purpose:**

The purpose of this study is to investigate the feasibility of applying openEHR (an archetype-based approach for electronic health records representation) to modeling data stored in EEGBase, a portal for experimental electroencephalography/event-related potential (EEG/ERP) data management. The study evaluates re-usage of existing openEHR archetypes and proposes a set of new archetypes together with the openEHR templates covering the domain. The main goals of the study are to (i) link existing EEGBase data/metadata and openEHR archetype structures and (ii) propose a new openEHR archetype set describing the EEG/ERP domain since this set of archetypes currently does not exist in public repositories.

**Methods:**

The main methodology is based on the determination of the concepts obtained from EEGBase experimental data and metadata that are expressible structurally by the openEHR reference model and semantically by openEHR archetypes. In addition, templates as the third openEHR resource allow us to define constraints over archetypes. Clinical Knowledge Manager (CKM), a public openEHR archetype repository, was searched for the archetypes matching the determined concepts. According to the search results, the archetypes already existing in CKM were applied and the archetypes not existing in the CKM were newly developed. openEHR archetypes support linkage to external terminologies. To increase semantic interoperability of the new archetypes, binding with the existing odML electrophysiological terminology was assured. Further, to increase structural interoperability, also other current solutions besides EEGBase were considered during the development phase. Finally, a set of templates using the selected archetypes was created to meet EEGBase requirements.

**Results:**

A set of eleven archetypes that encompassed the domain of experimental EEG/ERP measurements were identified. Of these, six were reused without changes, one was extended, and four were newly created. All archetypes were arranged in the templates reflecting the EEGBase metadata structure. A mechanism of odML terminology referencing was proposed to assure semantic interoperability of the archetypes. The openEHR approach was found to be useful not only for clinical purposes but also for experimental data modeling.

## 1 Introduction

The domain of neuroscience is currently one of the most progressive fields in health-care research (as witnessed in, e.g., the Horizon 2020 Societal Challenges list). Given that fact, the number of electroencephalography (EEG)/event-related potential (ERP) data resources that include data from clinical EEG/ERPs, experimental EEG/ERPs, neuro-rehabilitations, assistive systems based on EEG/ERPs, and household BCI (brain–computer interface) devices increases rapidly and necessitates stricter requirements on the data formats and storages used. Nevertheless, there is still an apparent lack of matured data formats and standards in the experimental as well as in the clinical EEG sphere. Moreover, many existing software solutions have been designed only for internal purposes of the user group in which the software solution has been developed.

The EEGBase portal (Ježek and Mouček, 2012) (developed at the University of West Bohemia) is a software tool focused mainly on the annotation, storage, management, and sharing of EEG/ERP (electroencephalography/event-related potential) experiments. Although EEGBase partially implements some existing standardization efforts and uses semantic web technologies, it is very closely related to EEG/ERP purposes only. This close relation imposes a limitation not only in case of stored experiment types but also in case of stored experiment metadata, which could be considered as stand-alone experiments (stand-alone medical reports). The main objective of this work is to utilize EEG/ERP health data characteristics to extend the potential of EEGBase interoperability and to increase the efficiency of the EEGBase metadata storage.

The vast majority of advanced health institutions archive medical health records electronically. However, these electronic health records (EHRs) are very often unstructured and, therefore, require a significant effort to facilitate machine readability. The free EHR structure influences not only its machine-readability and further computational analysis but also the exchange of records between institutions. The standardization of communication protocols and structured data models describing particular health domains increases overall data interoperability, unambiguity, and readiness for further analysis. Since these abilities are important for any kind of health data (not only for clinical data), a standardized data structure is considered to be useful also for experimental health data.

The standards supporting health data interoperability are based mainly on the (i) explicit description of data meaning, (ii) terminology and structure separation, and (iii) controlled vocabularies integration. The openEHR (Kalra et al., 2005) approach provides a multi-model, single source EHR framework. openEHR data models (so-called archetypes) stored in the openEHR CKM (Clinical Knowledge Manager) public repository could be used for medical data in general, not only for clinical purposes. While the CKM contains hundreds of archetypes describing many medical domains (suitable for metadata), but excluding the EEG itself, a set of new archetypes covering the EEG (EEG/ERP, respectively) domain is proposed in this work. These archetypes are derived from the EEGBase data/metadata structure as well as from other common well-known EEG data formats. The terminology is mainly based on a controlled vocabulary taken from the *odML terminology*.^1^

The article is organized as follows. The work context, existing common data format descriptions, and archetype development methods are described in Section 2. A set of archetypes and templates regarding the EEG/ERP domain is described in Section 3—Results. The final discussion is presented in Section 4.

## 2 Materials and Methods

### 2.1 Work Context

#### 2.1.1 EEGBase Data

A neuroinformatics laboratory specializing in the development of the software and hardware infrastructure (Mouček et al., 2014) for electrophysiology and in the analysis of EEG/ERP experiments was established at the University of West Bohemia under the Department of Computer Science and Engineering^2^ in 2003. EEGBase (Ježek and Mouček, 2012), a portal for experimental data management and sharing, was proposed and developed within this infrastructure. Since efficient data sharing is very closely related to used data structures and descriptions, EEGBase strongly emphasizes an effective separation of data and metadata and their storing in commonly known well-described formats.

**Figure 1:**
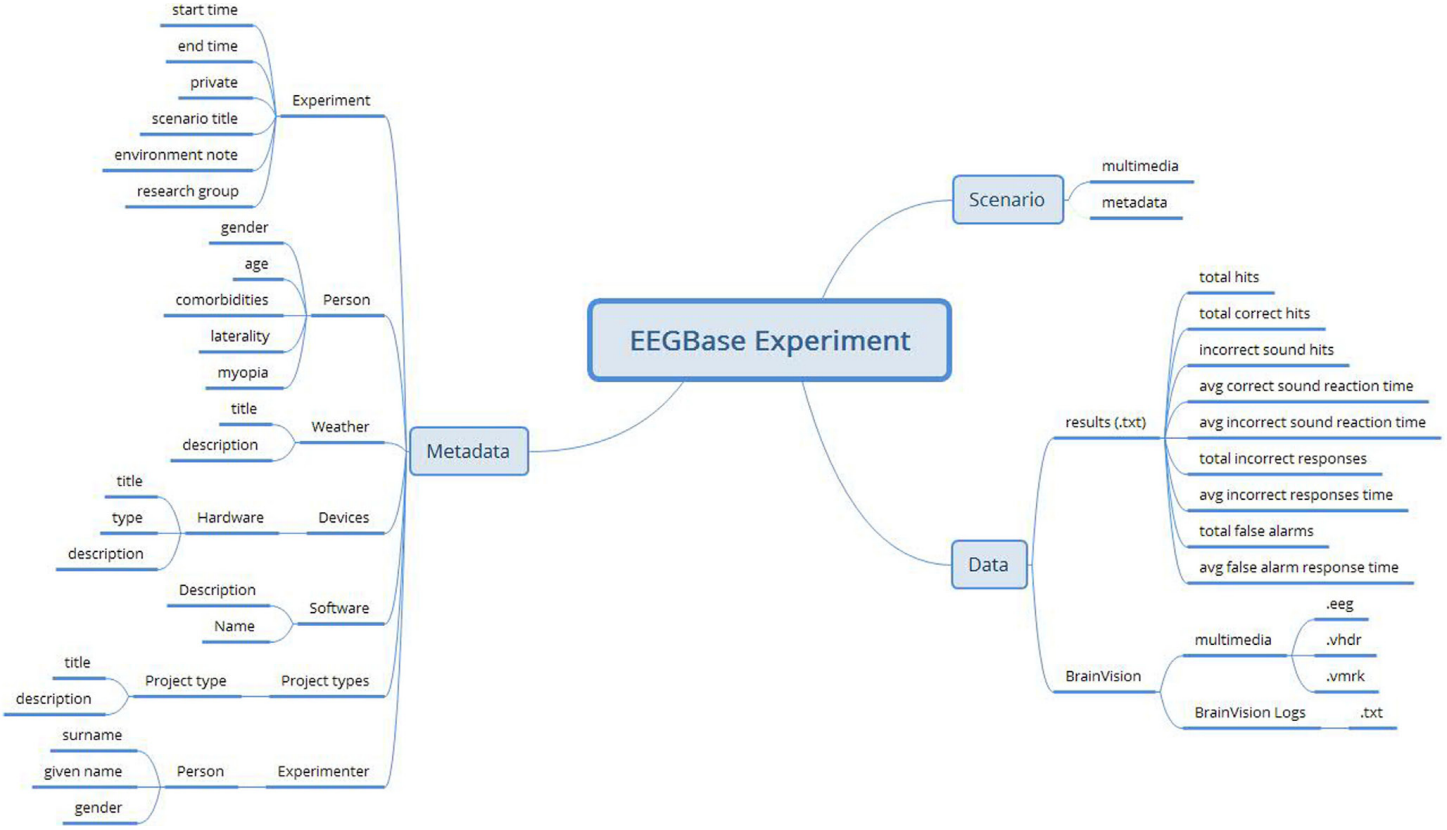
**An example of an EEGBase experiment structure consisting of the data, metadata, and scenario sections**. This example also illustrates that, even in such a relatively simple structure, there is no clearly defined border between data and all metadata.

The EEGBase data are stored in the BrainVision EEG format (BrainProducts, 2013), which is a proprietary solution of Brain Products GmbH but has an open specification. The EEGBase metadata related to the experiment is stored in the odML (open metaData Markup Language) (Grewe et al., 2011) structure and can be easily serialized into the XML format. In addition to the stored data and metadata, EEGBase also contains experimental scenarios describing the recording work-flow and used stimulation. The EEGBase scenarios are most often designed in the Presentation^®^ software tool (developed by Neurobehavioral Systems, Inc.^3^). However, since they vary from a flashing light to a prescripted computer game level, it is hard to predict the specific format a scenario is stored in. Therefore, EEGBase handles scenarios as multimedia attachments of the particular experiment. An example of an EEGBase experiment structure (from a project investigating developmental coordination disorders in children) is shown in Figure 1.

Common categories of metadata are structural and descriptive (sometimes also administrative, e.g., creation date, file type (NISOPress, 2004)). Structural metadata describes the structure in which data is stored (typically a table header) and descriptive metadata describes (or identifies) the nature and origin of data. In the case of EEG recordings, we can consider raw brain waveforms as data. The structure in which these data are stored (commonly binary data representation) is described by structural metadata. Descriptive metadata contains information about data acquisition and, in most cases, provides the knowledge necessary to provide the correct technical data interpretation, e.g., the number of electrodes, electrode impedance, or event time stamps. In the case of the BrainVision format, this metadata is included in the respective output files (.vmrk and .vhdr files). Unless stated otherwise, the metadata discussed is hereafter considered as descriptive.

Nevertheless, there is also metadata related to the whole experiment (environmental conditions, subject state, etc.), which is important for the context interpretation of the recording. EEGBase separates this metadata set and uses the odML structure and odML terminology for its description. Also, each scenario has its own metadata set related to the scenario raw data. These metadata sets are stored independently of each other (as they are related to different data) and cannot be easily aggregated. Moreover, if we consider an example when the EEG experiment is extended with a blood pressure (BP) measurement, then the BP measurement is a part of the metadata set related to the whole experiment, i.e., BP is included in the semantic context of the experiment. The BP measurement output is defined by two numerical values representing the (i) systolic and (ii) diastolic blood pressure. Both values (data) have their metadata: e.g., units (millimeter of mercury), measurement date and time, or recording device. Even though the BP data and metadata are a part of the EEG experiment metadata set, it is also an autonomous fully fledged measurement output. This example shows that the definition of what data and metadata are and where the border between data and metadata is, is a matter of perspective.

Another view on metadata should be mentioned. If data are considered as a potential analysis input, then metadata could be considered an analysis input filter and/or an analysis parameter. The BP data and metadata from the previous example could be analyzed separately or could serve as an input filter for the next EEG/ERP data analysis. Since EEG/ERP and BP recordings are logically separated for the next analysis, they have to be also separated structurally to achieve an efficient computational process. (Deeply embedded BP recording data in the EEG/ERP experiment metadata structure would be hard to process independently.) The openEHR approach is designed to model various measurements/observations separately and link them together.

#### 2.1.2 Electronic Health Records and openEHR

Since most data and metadata collected during experimental work in the EEG/ERP domain could be classified as health data, it is beneficial to apply some recommendations and frameworks, designed primarily for clinical health data and electronic health records (EHR), to them:

- a unified and explicitly defined concept controlled by higher authorities,
- a unified data description regardless of the institution in which the data have been acquired (e.g., in a hospital or experimental laboratory),
- knowledge of the concept that makes data sharing simplified and data interpretation unambiguous,
- predefined data structures validated by domain experts in a ready-to-use form that could be used within the data structures describing the EEG/ERP experiment (e.g., the BP example),
- an implicit logical separation of data and metadata,
- analytical tools and storage solutions driven by a unified concept,
- a middle-ware for potential mapping between existing formats.

When describing EHR standards/architectural patterns/frameworks, the following important representatives should be mentioned:

1. HL7 (Dolin et al., 2006) is a set of international data transfer standards, guidelines, and methodologies. The fundamental components are conceptual standards (e.g., Reference Information Model), document standards (e.g., Clinical Document Architecture), messaging standards, and application standards (e.g., Clinical Context Management Specification).
2. CEN/ISO 13606 (Muñoz et al., 2011) is a European standard defining an information architecture of EHR. It is based on generic predefined information models. The architecture is proposed to be mapped to HL7 v3.
3. openEHR is an open standard, which provides two-layer modeling methodology in which the general concept is formed by a generic reference model (RM) and the specific domain or its part is formulated by archetypes. It is based on ISO 13606 and its RMs describe demographics, services, clinical content, and clinical work flows.

HL7 is mainly designed for data transfer between institutions, while CEN/ISO 13606 and openEHR are focused on the explicit specification of clinical content and work flow. Moreover, their design is proposed also for user-centric systems (unlike HL7). The openEHR concept and features could be helpful in the context of problems related to data/metadata separation.

openEHR is an open domain-driven platform for developing flexible e-health systems. This platform, with respect to ISO/CEN 13606, presents a set of generic RMs as an abstract specification of elements/processes in the health sphere (e.g., *Observation, Action, Instruction*, or *Evaluation*). Above the RMs, so-called archetypes, abstract representations describing particular domains or their parts, are modeled (e.g., ECG or blood pressure). While RM is only an abstract model, which can be expressed, e.g., by UML (Unified Modeling Language), openEHR archetypes can be expressed in the machine-readable Archetype Definition Language (ADL). The archetype structure consists of five mandatory sections: archetype ID, concept, language, definition, and ontology and four optional sections: specialization, description, invariants, and revision history. While some sections represent an archetype metadata set, the core of the archetype is specified in *definition* and *ontology* sections. The *definition* section describes the domain the archetype is focused on. Each attribute (so-called datapoint in the openEHR terminology) is denoted by its internal code/IDs. These IDs are paired with their real names, binding codes, and definitions in the ontology part. The binding sub-part refers to the terms used from an external resource (ontology, terminology, etc.), typically SNOMED CT (Stearns et al., 2001) or *odML terminology* in the case of this work. The revision history part of the archetype keeps the archetype metadata dealing with the changes in structure of a once-approved archetype. Each archetype has its life-cycle state specified in its metadata set (the whole set of states is defined by openEHR) according to its position in the publishing process.

Archetypes should be designed in a general way to enhance their re-usability. Afterward, the archetypes are usually implemented via templates, the third layer of the openEHR multilayer approach, which allow users to define more restrictions, connect two or more archetypes together, and reduce the datapoint set of the archetype. To maximize re-usability of the existing archetypes, public repositories called Clinical Knowledge Managers (CKMs)^4^ have been built. Since no EEG archetype has existed in the CKM so far, there was an opportunity to create it.

Even though openEHR is not matured in some ways (e.g., in the quality of development tools), it has been successfully deployed at various places in the UK, Australia, and Russia. Moreover, with respect to its active community and openness, the framework has a chance of becoming more widespread in the future.

### 2.2 Descriptions and Formats in Electrophysiology

To create a new EEG openEHR archetype set, the current EEG format and domain standardization efforts have been investigated. Although there is no common and widely accepted universal format for electrophysiology data/metadata, the current standardization initiatives/efforts and their important projects and outcomes are presented and taken into consideration during the archetype development. The purpose of this work is not to compete with the existing proposals and solutions, but to be in line with them and their current potentials.

#### 2.2.1 Open metaData Markup Language

The open metadata Markup Language (odML (Grewe et al., 2011)), developed by G-Node (German Neuroinformatics Node), is an explicit specification of the metadata exchange format, which is generic enough to store textual metadata from any scientific discipline. The model allows the construction of a tree-like structure from the *Sections*. For each *Section*, a set of properties and values can be defined, as shown in Figure 6. In addition to this generic format, the *odML terminology* for electrophysiology was established. The usage of the odML structure, together with the terminology for electrophysiology, provides a machine-readable metadata set limiting the ambiguity of the used terms.

#### 2.2.2 NIX Format

While odML is focused on the metadata exchange, NIX (Stoewer et al., 2014), G-Node’s follow-up project, also solves the related data storage. NIX defines a generic data model to represent data and metadata with flexible back-ends and provides an open standardized data format. The current authors’ implementation combines the odML structure for metadata and the HDF5 (Folk et al., 2011) container for data, i.e., NIX defines a standard schema for HDF5 files to represent the generic model. The basic NIX structure defines data (typically time series) as N-dimensional *DataArray*s. Each *DataArray* is related to its data *Source* (e.g., the channel specification) and has its *Dimension*. The data can be annotated by *Tag*s to define its specific parts (e.g., time points or time intervals). All parts of the structure can also be annotated by additional odML metadata sections.

#### 2.2.3 Neurodata without Borders Data Format

The increasing popularity of HDF5 is also reflected in the Neurodata Without Borders (NWB) data format. The NWB project established a new data format based just on HDF5. The NWB data format defines detailed data model, which is much stricter than the more generic one in NIX (Teeters et al., 2015). NWB as a stand-alone format was proposed to avoid an additional mapping layer between NWB strict models and current solutions like NIX. Since NWB is still in its early phase of development, it will not be further considered.

#### 2.2.4 Ontology for Experimental Neurophysiology

Ontology for Experimental Neurophysiology (Le Franc et al., 2014) (OEN) provides a formal explicit representation of experimental neurophysiological data/metadata. The OEN development was initialized under the INCF Program on Standards for Data Sharing as the answer to insufficient ontological resources in the domain. The main OEN purpose is to provide a controlled vocabulary to standardize descriptions of domain resources. OEN is also in its early development phase.

#### 2.2.5 EEG BrainVision Data Format

The EEG BrainVision data format developed by Brain Products GmbH^5^ organizes raw data and metadata from recordings into three files: (i) a textual (*INI files-like* format) header file containing descriptive metadata, (ii) a textual (*INI files-like* format) marker file containing event timestamps, and (iii) a binary representation of raw brain waveforms. The header file describes mainly the technical metadata: e.g., the number of channels, channel resolution, data format, information about binary/ASCII data representation, or sampling interval. The metadata related to the subject or experiment itself is not included.

#### 2.2.6 European Data Format

The European Data Format (EDF) and its extension EDF+ is another standardization effort and data format for EEG, sleep recording, electrocardiography (ECG), electromyography (EMG), and evoked potentials. EDF+ can save annotations and analysis results (Kemp et al., 1992; Kemp and Olivan, 2003). Just as the BrainVision format, EDF+ is strictly focused on the recording itself. The EDF+ format organizes the content into *Header records* and *Data records* sections. The *Header records* section includes both file and recording metadata—e.g., the data format version, patient’s ID, start time of recording, or number of channels in recording. The *Data records* section contains consecutive data for all channels.

### 2.3 Design of EEG Archetypes and Templates

The design procedure of a basic EEG archetype set can be described in the following steps:

1. the concepts and their sub-parts are determined within the EEGBase experiment structure, NIX structure, and EDF+ attributes,
2. the same and/or similar concepts are aggregated,
3. the redundant and unimportant attributes are eliminated,
4. the determined concepts and their semantically most suitable openEHR RMs are matched (when this is not possible, the concept is separated into smaller pieces and matched with the RM Cluster),
5. the archetypes corresponding to the determined concepts are implemented in ADL,
6. the archetypes are bound with external terminology,
7. the templates covering the EEGBase experiment structure are implemented.

#### 2.3.1 Concepts Determination

**Figure 2:**
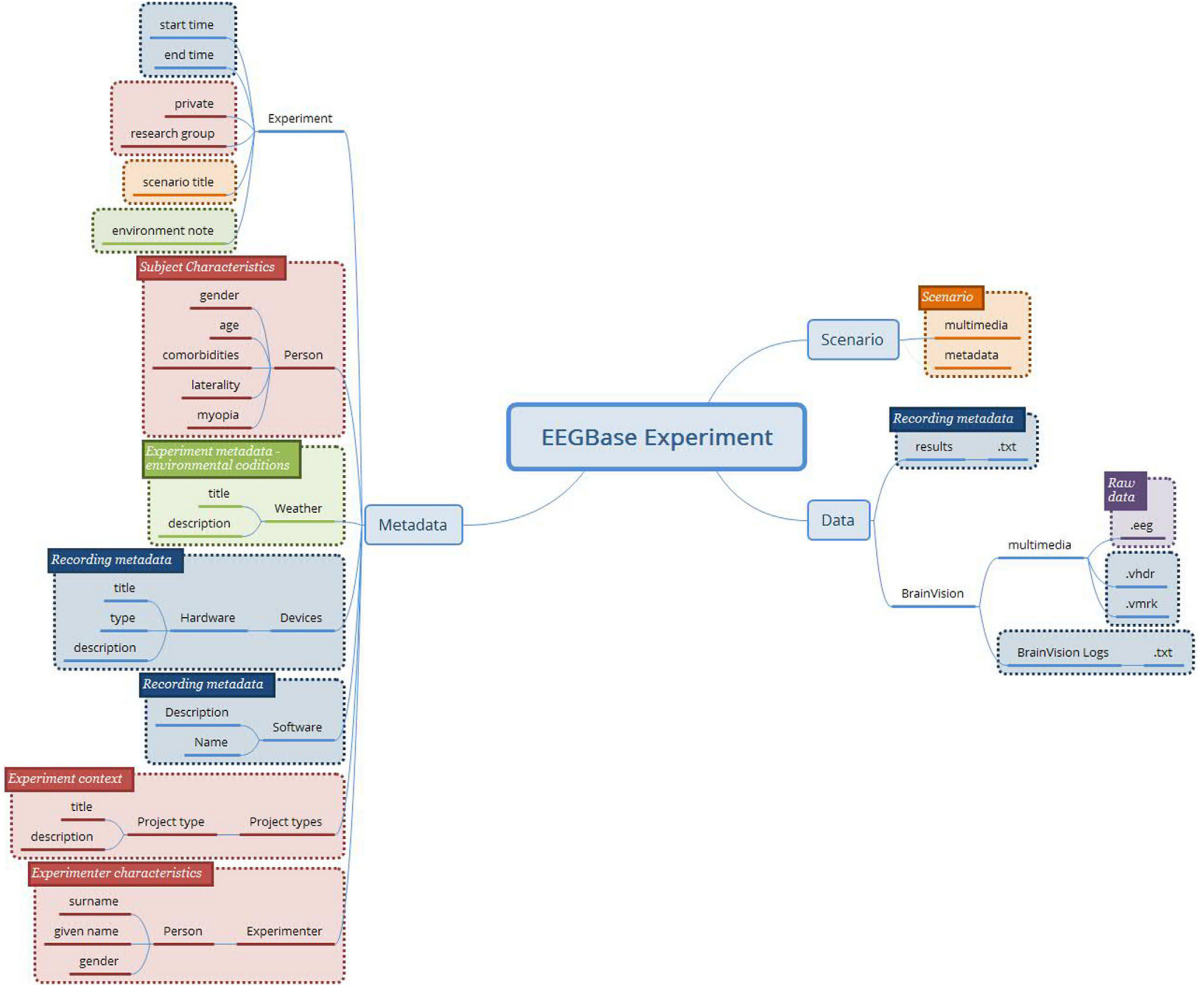
**The concepts determined from the EEGBase experiment structure (blue boxes—metadata related to the recording itself; purple box—the binary file representing raw brain waveforms; orange boxes—data and metadata related to the experiment/trial scenario; green boxes—the experiment metadata; red boxes—the context of the experiment and the description of the tested subject)**.

Figure 2 shows an example of the concepts determined from the EEGBase experiment structure. Since the experiment context is superior to the experiment itself from the hierarchical perspective, it should be a responsibility of the system which implements the archetypes to link it properly with external archetypes/data (e.g., subject’s/experimenter’s demographical information or information related to the project are not described inside the new archetypes). The subject’s overall characteristics and state were composed from other health records, which were not directly connected to the EEG experiment. Therefore, these red boxes are not further considered in the archetypes design. The NIX and EDF+ attributes were processed in the same way.

#### 2.3.2 Concepts Aggregation and Attributes Elimination

During this phase, the attributes of the same concepts, but of various data representations (EEGBase, NIX, EDF+), were aggregated. The following rules were applied:

- if two or more attributes are identical, then only one is kept,
- single attributes are kept,
- if two or more attributes are semantically identical (i.e., they have the same meaning but different representation), then only one is kept following the resource priority: 1. NIX; 2. EEGBase; 3. EDF+
- unimportant attributes or attributes with no explicit meaning (e.g., the attributes related to specific resource structures like IDs) are removed.

**Figure 3:**
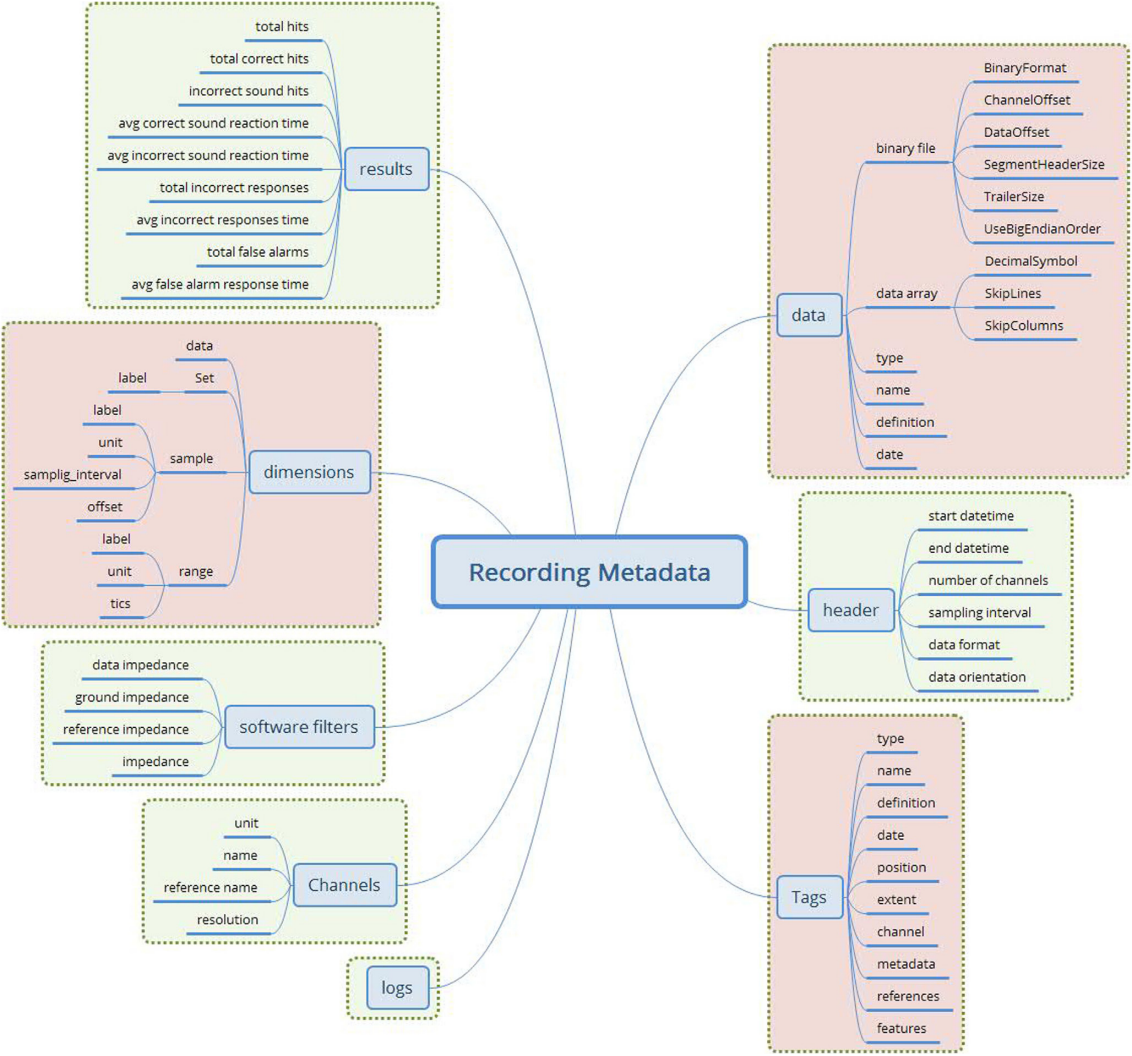
**The merged recording metadata concept (green—EEGBase/BrainVision attributes; red—NIX attributes)**. This metadata set contains the attributes taken from the EEGBase and NIX structures. The attributes are aggregated according to the predefined set of rules. No attribute from EDF+ is used.

The final attribute set is shown in Figure 3.

#### 2.3.3 Matching Concepts with openEHR Reference Models

**Figure 4:**
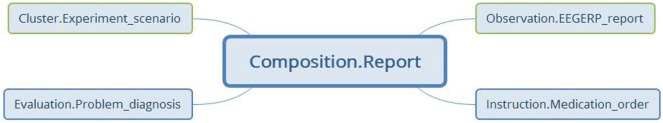
**The newly proposed (green) and existing (blue) archetypes connected into *Composition* RM**.

When the processes of the concepts determination and aggregation were completed, the matching of concepts characteristics with the characteristics of openEHR RMs was necessary. openEHR presents five RMs representing the medical entry types (*Observation, Evaluation, Action, Instruction*, and *Administrative Entry*), two RMs expressing the entry structure (*Composition* and *Section*) and three RMs characterizing the data structure (*Element, Cluster*, and *Structure*). Because of the evident EEGBase metadata diversity (recording metadata, experiment metadata, scenario metadata, etc.), the final model had to be composed of multiple archetypes. As an alternative to a data container, openEHR provides so-called RM *Composition*; a *Report Composition* archetype exists in the current CKM. This archetype is perfectly suitable also for EEG/ERP experiment data and metadata. The following archetypes are then connected to the *Report* archetype via an inner reference solution—a so-called slot (Figure 4).

1. Problem/Diagnosis (Evaluation RM)
2. Medication order (Instruction RM)
3. Experiment scenario (Cluster RM)
4. EEG/ERP Result (Observation RM).

The *Problem/Diagnosis* archetype (*Evaluation RM*) that already exists in the CKM was included for the specification of various diagnoses, which are closely related to the EEG/ERP experiment itself and not to the subject’s overall health status (e.g., sleep deprivation induced for purposes of the experiment).

The *Medication order* archetype (*Instruction* RM) that is also stored in CKM serves for the description of the medication or other substances that are prescribed/administered to the subject for the purpose of the experiment only, i.e., it excludes the medication prescribed to the subject for any other reasons, the long-term medication included.

The *Cluster* RM archetype is the simplest archetype structure that serves for the tree-like data structure description without any reference to a specific clinical work flow. Since the *Experiment scenario* has no semantic nor structural relations to other specific RMs (*Medical Entry* RMs), it is designed as a *cluster*.

**Figure 5:**
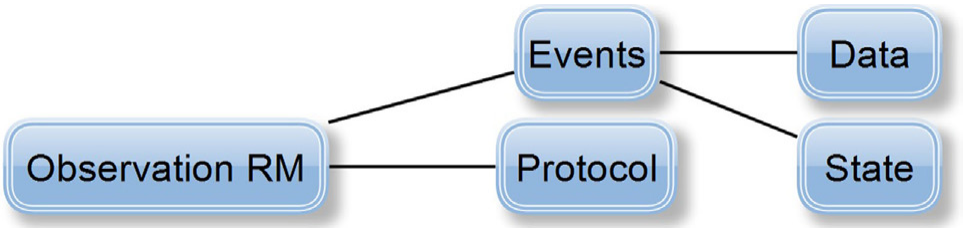
**Definition of *Observation* RM (Beale, 2016): *protocol* attribute describes the way the observation was realized (e.g., used hardware/software)**. *Events* attribute represents the series of time events during the observation, i.e., a set of events represents the observation history. Each event has its *Data* attribute, i.e., the observation results, and its *State* attribute describing the subject’s state related to the observation (e.g., the subject’s position—standing/sitting).

Finally, data acquisition, i.e., data and recording metadata, corresponds to the *data* and *protocol* part of the *Observation* RM (its characteristics and structure can be seen in Figure 5). The *Observation* RM also covers information about time events and the subject’s state (it differs from the *Problem/Diagnosis* archetype). Therefore, the *EEG/ERP Result* archetype could include the whole recording concept and become the most complex of all the proposed archetypes.

Besides the selected concepts and archetypes corresponding to them, two more archetypes describing *Software* and *Stimulus* (also missing in the CKM) were determined as necessary. The base for these archetypes was taken from the odML electrophysiological terminology.

**Figure 6:**
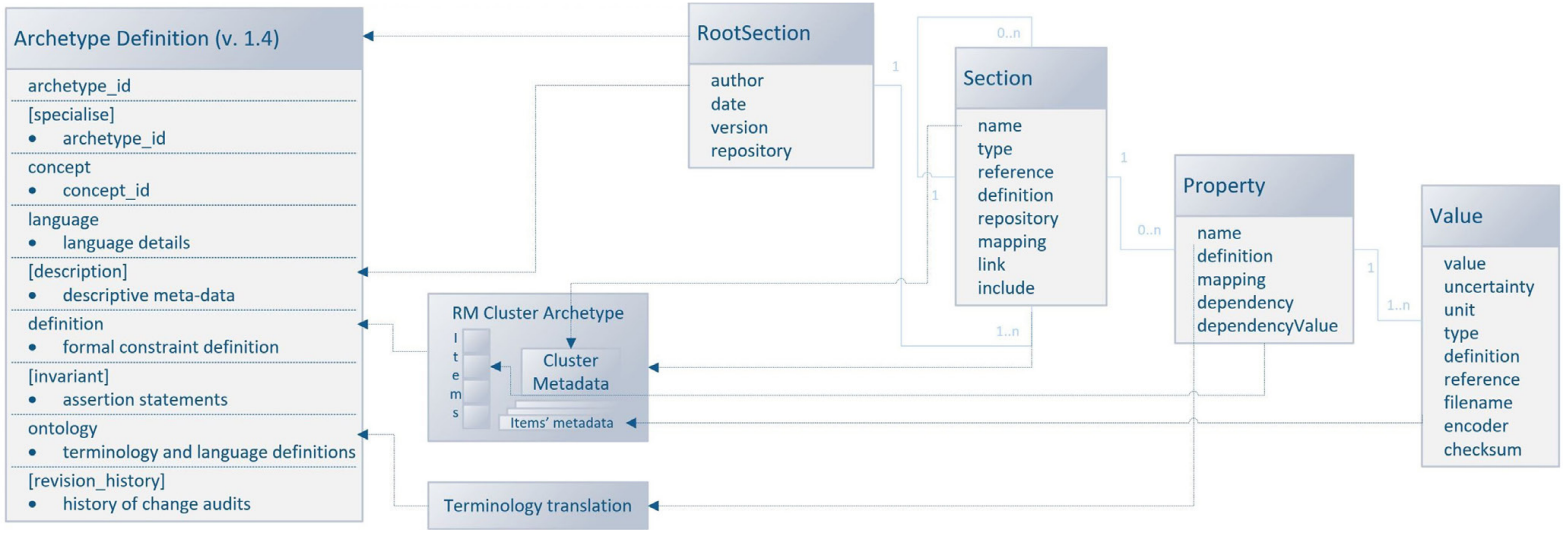
**Mapping of the odML structure to the openEHR archetype based on *Cluster* RM**. The left side of the figure shows the archetype sections: the sections in square brackets are optional, and the sections without brackets are mandatory. The right side of the figure shows the odML data model (Grewe et al., 2011) with relations (the light blue lines) between odML elements. The dashed lines show the relations/transformation rules between models. The middle part of the figure refers to the additional steps necessary for the transformation.

Since the odML structure can be expressed in a tree-like form, the *Cluster* RM could be used for any odML structure without any loss of expressive power. Figure 6 displays the transformation rules (dotted lines) and transformations of odML sections into openEHR clusters. The odML structure manipulates four basic elements: the *root section, section, property*, and *value*. Each part also has its attribute set. The root section represents one archetype and its attribute set was directly mapped to the archetype metadata set. Each odML section/subsection can be represented as a datapoint of the type cluster/nested cluster in the archetype body. OdML *Section* attributes were transformed into cluster metadata. Datapoints were then constructed from odML properties and their values specify, e.g., data types, definitions, units, or enumerations. Enumerations were transformed into the archetype body as predefined internal codes of the datapoints. While odML has no predefined datatype set, the datatypes used were manually compared and paired (Table 2) with the openEHR datatype set (according to the *odML terminology* data meaning and nature).

The mapping could be used for any odML “sections to cluster” archetype transformation. Therefore, the possibility of the further extension of the EEG/ERP archetype composition according to odML sections is ensured. Since the odML format is used for NIX metadata as well as for EEGBase experiment metadata, there is a possibility that new archetypes will be needed in the future. As the odML terminology is machine-readable, the proposed mapping could be used for the development of a tool for automatic odML to ADL transformation. All newly proposed archetypes are presented in detail in Section 3.

#### 2.3.4 Binding Archetypes with External Terminologies

It is also critical to bind archetype datapoints with external terminologies. The *odML terminology for electrophysiology* represents a comprehensive controlled vocabulary. Furthermore, since odML is used within NIX and EEGBase and parts of the terminology serve as a base for recently proposed archetypes (described in Section 3), it is suitable to bind archetype terms to the odML terminology. However, the odML terms have no explicitly defined referenceable (and dereferenceable) IDs. As the terminology is stored in a public repository (see text footnote 1) as a set of XML files, a particular term (the XML element the term is kept in) of the *odML terminology* can be referenced. This reference can be labeled with a unique ID. These IDs/pointers were created in four steps:

1. the file containing the root section corresponding to the particular concept was localized,
2. the XPath (the language for addressing parts of an XML document (Clark and DeRose, 1999)) query to the specific XML element containing the searched term was constructed,
3. the paths obtained in step 1 and 2 were aggregated into one string,
4. a short unique alias (that serves as an identifier to the string created in step 3) was assigned.

As a running example, the term “Reference,” representing a reference electrode, was selected. The *reference electrode* term is located (from the structural point of view) in the *Electrode* section with the property *Usage* and value *Reference*.

All odML terminology XML files are available in a public repository and accessible via the Uniform Resource Locator (URL). In our example, the URL pointing to the file containing the *Electrode* section is shown in Listing 1.

**Listing 1**. The URL to the Electrode section.

http://portal.g-node.org/odml/terminologies/v1.0/electrode/electrode.xml.

Then the pointer to the reference electrode can be expressed as shown in Listing 2, where the hash separates the section name *Electrode* from the file URL. The colon separates the property name *Usage* from the section name and a slash separates the property name *Usage* from the enumerated item.

**Listing 2**. Step 1: Pointer to the Reference term in the Electrode odML section.

http://portal.g-node.org/odml/terminologies/v1.0/electrode/electrode.xml#Electrode:Usage/Reference.

**Figure 7:**
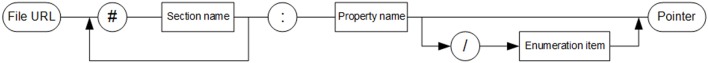
**The syntax diagram describing the pointer string construction**.

The diagram in Figure 7 shows the pointer string construction.

In the second step, an XPath string querying the element with the searched term from the selected XML file is constructed. Listing 3 shows the XPath query for the running example.

**Listing 3**. Step 2: The XPath query for the term Reference within the electrode odML section.

odML[@version = "1"]/section/property/name[text() = Üsage"]/../value[text() = "Reference"]

Within the third step, the XPath query (step 2) and the pointer string (step 1) are merged together. The final string (Listing 4) contains the path to the particular file and the XPath query to the particular element. Thus, the pointer string can be automatically dereferenced by a machine.

**Listing 4**. Step 3: The merged pointer string and XPath query.

http://portal.g-node.org/odml/terminologies/v1.0/electrode/electrode.xml#/odML[@version = "1"]/section/property/name[text() = Üsage"]/../value[text() = "Reference"].

The string from step 3 is not suitable for direct usage inside the archetype. When this string is used as an external terminology code inside the binding section of the archetype, existing tools and libraries like the *Ocean Informatics Archetype Editor* or openEHR java libraries evaluate the archetype as invalid. Therefore, the aliases were created. Each alias consists of the prefix ODMLID, a 3-digit section number and a 3-digit term number. The list of aliases, section numbers, XPath queries, pointer strings, etc., are kept in a separate table, which is a supplemental resource for the archetypes and templates. In our running example, the alias bounded with the particular internal archetype code is ODMLID007013.

## 3 Results

### 3.1 EEG/ERP Result Archetype

**Figure 8:**
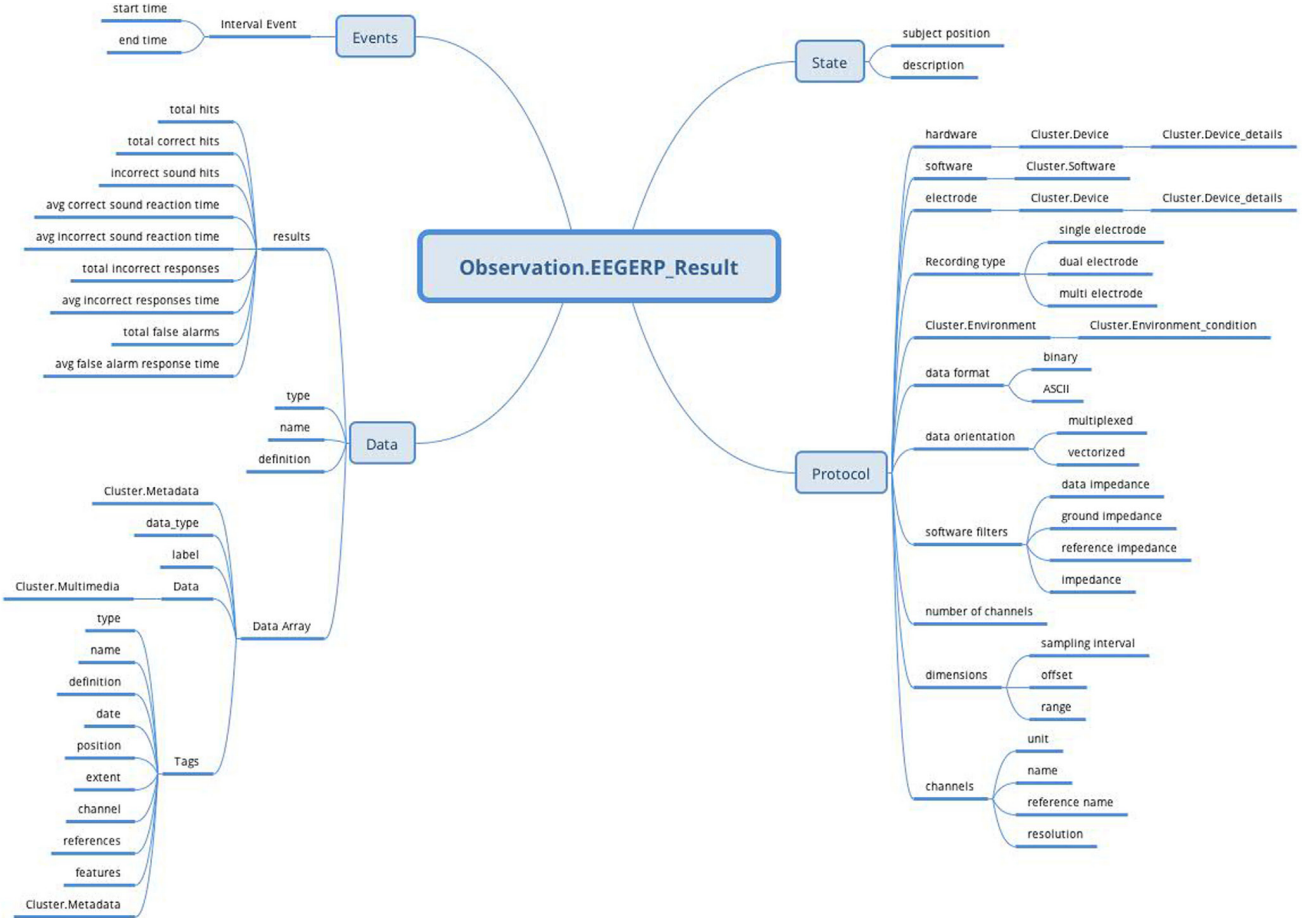
***EEG/ERP Result* archetype (*Observation* RM) structure: the archetype contains all attributes (*Protocol, Data, Events*, and *State*) supported by *Observation* RM to cover recording conditions, results, time specifications, and subject’s position**.

The *EEG/ERP Result* archetype is based on the *Observation* RM, covers the EEG recording, and uses the attributes from NIX, EEGBase (the BrainVision EEG format) and EDF+. These attributes are divided into *data* and *protocol* parts and turned into the archetype’s datapoints. Figure 8 shows the final archetype structure.

**Figure 9:**
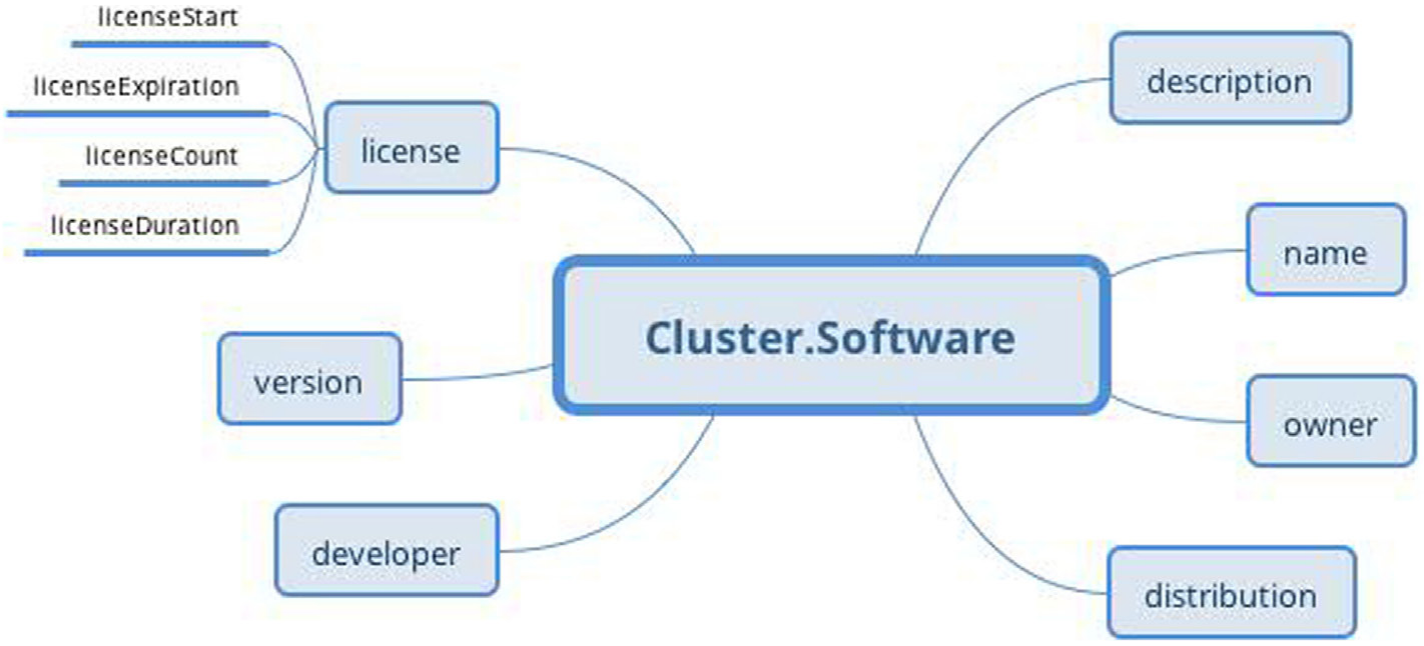
***Software* archetype (*Cluster* RM) structure: the archetype contains basic software information identical to that of the odML terminology for electrophysiology**.

The protocol part describes the metadata important for data interpretation and recording reproduction. The protocol describes recording conditions and the way the recording was conducted. The *Hardware* and *Electrode* slots are connected with the *Device* archetype (designed by openEHR developers together with the *Device details* archetype) and describe the used hardware and electrodes, respectively. The *Software* slot is connected to the newly created archetype Software of the type *Cluster*. The structure of the *Software* archetype is taken from the odML terminology for electrophysiology section of the identical name (Figure 9). Finally, the *Environment* slot is connected to the *Environment* archetype existing in the CKM. Crucial cardinalities (e.g., if at least one electrode per recording is required) are explicitly specified in the archetype body.

The *Person*’*s state* part is natively supported by the *Observation* RM. The state is restricted only to the subject’s position and textual description. The complex subject’s state characteristics can be expressed by the *Problem/Diagnosis* archetype of the *Evaluation* RM within the same *Report Composition*.

The *Events* part, also natively supported by the Observation RM, represents events as time intervals when the recording was done.

Finally, the *Data* part is composed of two major branches. The *Data Array* branch allows the storage of binary data (e.g., in a BrainVision.eeg file) as complex structures compatible with NIX. The datapoint structure is designed primarily according to NIX and extended with some EEGBase (BrainVision) attributes. The *Results* branch provides a summary of recording results. As this part is based on the EEGBase experiment attributes, it contains the attributes closely related to the EEG/ERP experiments only (e.g., statistics of stimuli events).

### 3.2 Experiment Scenario Archetype

**Figure 10:**
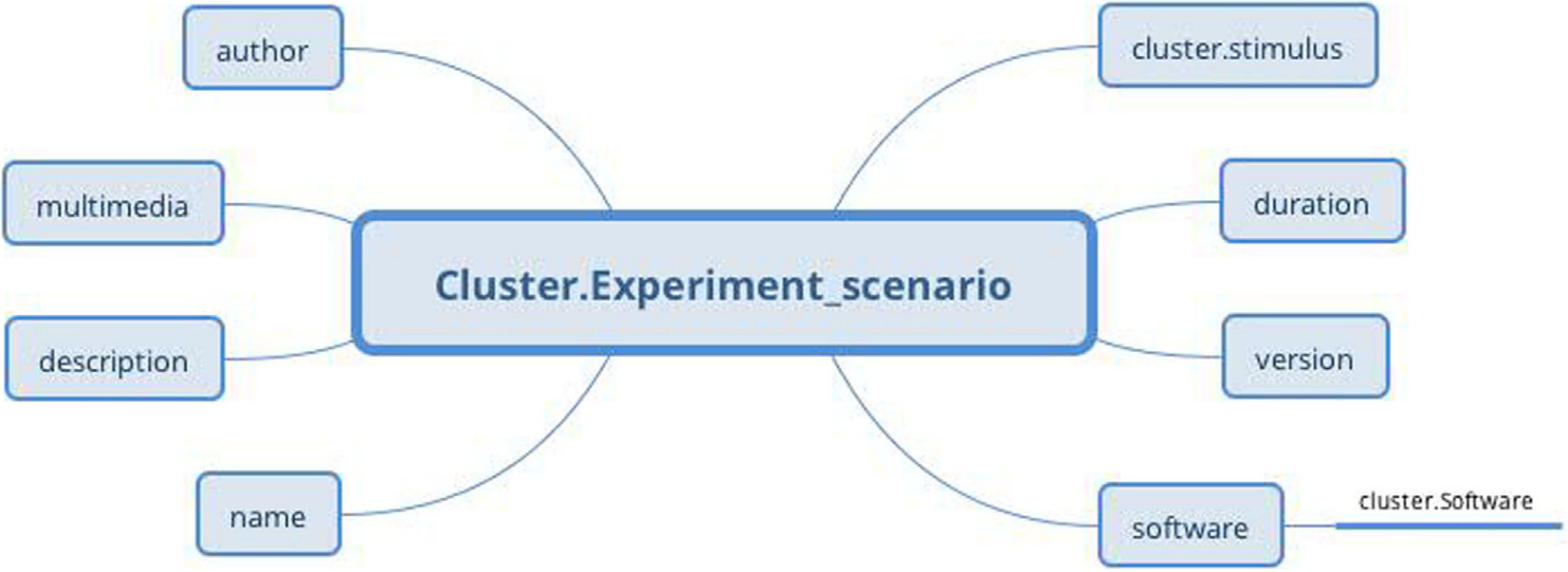
***Experiment scenario* archetype (*Cluster* RM) structure: the archetype contains general information about the scenario, the original scenario files as a multimedia attachment, information about software in which the scenario was built (via *Software* archetype), and information about used stimulus, if needed (via *Stimulus* archetype)**.

The *Experiment scenario* archetype (Figure 10) based on the RM *Cluster* is derived from the EEGBase experiment attributes dealing with a scenario description (Figure 9). The *stimulus* slot is connected to the *Stimulus* archetype (not presented for the huge amount of datapoints), which is derived from the odML section of the identical name. While odML distinguishes between various stimuli types, the archetype aggregates all datapoints into one structure and a particular stimulus can be determined using templates (see Section 3.5). Table 1 presents a list of all used and proposed archetypes.

**Table 1:** **List of archetypes covering the EEG/ERP domain**.

Archetype	Reference model	Status
Report	Composition	CKM
Medication order	Instruction	CKM
Problem/diagnosis	Evaluation	CKM
EEG/ERP result	Observation	New
Experiment scenario	Cluster	New
Software	Cluster	New
Stimulus	Cluster	New
Device	Cluster	CKM
Environmental conditions	Cluster	CKM

**Table 2:** **OdML and openEHR datatypes pairs**.

odML terminology	openEHR datatype	Notes
Person	N/A	Slot for the demographic archetype instances
Date	date time	
Text	text	
Int	count	
String	text	
Float	quantity	
Binary	multimedia	
URL	URI	
Datetime	date time	
Time	date time	In case of start and end time presence at once, those times are merged in an *interval* of datetimes
Boolean	Boolean	
2-tuple	N/A	Substituted by cluster containing two text/quantity/count types

### 3.3 Problem/Diagnosis Archetype

The *Problem/Diagnosis* archetype based on the *Evaluation* RM is used as published in the CKM. The archetype is designed to describe a single health issue that impacts the physical, mental, and/or social well-being of the subject. In the EEG/ERP experiment, it allows us to specify the experimental condition of the subject only. While health issues are described in an unstructured format, additional information (e.g., date/time of resolution, severity) is structured.

### 3.4 Medication Order Archetype

The *Medication order* archetype based on the *Instruction* RM is used as published in the CKM. The archetype is designed to describe a single item administered to the subject. In the EEG/ERP experiment, it allows us to specify the medications given to the subject for the purpose of the experiment only. Besides the item identification, the archetype allows us to specify the medication order in more than 20 datapoints.

### 3.5 openEHR Templates

Since archetypes are mainly used via templates, some testing templates covering the selected types of EEG/ERP experiments were created. Since these templates are too large to visualize, no figure example is provided.

The templates focusing directly on stimuli were proposed according to the odML terminology. These templates reduce the original set of the *Stimulus* archetype datapoints to a subset according to the particular stimulus type characteristics (e.g., movie, pulse, or ramp).

## 4 Discussion

The main EEGBase constraint is the strict separation of experiment data and experiment metadata. This feature may cause serious difficulties with the further extension of the metadata set, especially by complex structures taken from other measurements (e.g., blood pressure measurement). The main aim of this work was to investigate the potential benefits of openEHR principles applied to EEGBase. The investigation has shown that the EEGBase experiment data could be mapped as a composition of newly proposed and already existing archetypes. Moreover, the archetypes have been developed inline with the already existing EEG formats (NIX, EDF+, and the BrainVision EEG format), respecting the odML terminology. Therefore, the data stored in these structures could be easily mapped into our archetype structures. The proposed mapping allows us to present the existing data in the form that is typical for the health domain/health records. Apart from the created archetypes and templates, an algorithm for referencing/dereferencing odML terms was proposed.

The archetype publication process (i.e., the process during which the proposed archetype goes through various life cycle phases such as *Author*’*s draft, Team reviewed*, and *Published*) is currently in progress and an incubator for the archetypes (the place where the archetypes are accessible to domain experts for additional comments and modifications) was created within the official openEHR CKM.

Currently, there is no specific deployment of the proposed archetypes. The implementation of the whole archetype concept into the EEGBase portal is a challenge from the architectural point of view. The concept of a personal EHR system (Papež and Mouček, 2015) based on openEHR (currently under the development of our research group) implements the EEG/ERP archetypes.

The key benefit of this personal EHR system and/or improved EEGBase is in the granularity of experiment data/metadata and in the existence of the flexible border between data and metadata. The system design allows researchers to choose which dataset can be used as data (the analysis input) and which dataset can be used as metadata (analysis attributes and filters). This approach also enables researchers to do a reverse analysis; e.g., the blood pressure could be analyzed according to the body mass index (BMI) criteria or the BMI could be analyzed according to the blood pressure criteria. Furthermore, all datasets could be processed using the same software tools (e.g., the tools based on openEHR Java libs). It allows researchers to focus on the data itself and not on the data extraction process. The potential use of the developed EEG/ERP archetypes in the clinical domain is beyond the scope of this article.

Given the fact that openEHR respects ISO/CEN 13606 and that the translation rules between HL7v3 and openEHR exist, the created archetypes can be transformed to HL7v3 models/ISO/CEN 13606 archetypes and serve in the EHR systems based on HL7v3/ISO/CEN 13606. Together with the completion of the publication process, these domain transformations will be performed in the future. The current gap between experimental and clinical data descriptions could be reduced.

## Author Contributions

VP designed the study and wrote the manuscript. RM supervised the project and commented on the manuscript.

## Conflict of Interest Statement

The authors declare that the research was conducted in the absence of any commercial or financial relationships that could be construed as a potential conflict of interest.

## Footnotes

^1^https://github.com/G-Node/odml-terminologies.

^2^http://neuroinformatics.kiv.zcu.cz/.

^3^http://www.neurobs.com/.

^4^http://www.openehr.org/ckm/.

^5^http://www.brainproducts.com/.
